# TRIM67 Deficiency Exacerbates Hypothalamic Inflammation and Fat Accumulation in Obese Mice

**DOI:** 10.3390/ijms23169438

**Published:** 2022-08-21

**Authors:** Lanlan Jia, Zhengli Chen, Ting Pan, Yu Xia, Junbo He, Asad Jahangir, Xiaoli Wei, Wentao Liu, Riyi Shi, Chao Huang, Qihui Luo

**Affiliations:** 1 Laboratory of Experimental Animal Disease Model, College of Veterinary Medicine, Sichuan Agricultural University, Chengdu 611130, China; 2 Key Laboratory of Animal Disease and Human Health of Sichuan Province, College of Veterinary Medicine, Chengdu 611130, China; 3 Department of Basic Medical Sciences, Center for Paralysis Research, College of Veterinary Medicine, Purdue University, West Lafayette, IN 47907, USA

**Keywords:** TRIM67, hypothalamus, obesity, energy homeostasis, neuroinflammation

## Abstract

Obesity has achieved the appearance of a global epidemic and is a serious cause for concern. The hypothalamus, as the central regulator of energy homeostasis, plays a critical role in regulating food intake and energy expenditure. In this study, we show that TRIM67 in the hypothalamus was responsive to body-energy homeostasis whilst a deficiency of TRIM67 exacerbated metabolic disorders in high-fat-diet-induced obese mice. We found exacerbated neuroinflammation and apoptosis in the hypothalamus of obese TRIM67 KO mice. We also found reduced BDNF in the hypothalamus, which affected the fat sympathetic nervous system innervation and contributed to lipid accumulation in adipose tissue under high-fat-diet exposure. In this study, we reveal potential implications between TRIM67 and the hypothalamic function responding to energy overuptake as well as a consideration for the therapeutic diagnosis of obesity.

## 1. Introduction

The global obesity epidemic continues its relentless advance, currently affecting > 2 billion people or ~30% of the population of the world [1]. Obesity not only increases the risk of a number of metabolic diseases, including type 2 diabetes, but also the risk of dementia such as Alzheimer’s disease (AD) [2]. The imbalance between food intake and energy expenditure is the culprit. The hypothalamus, which controls a number of neuroendocrine functions integrating metabolic feedback and regulating energy homeostasis [3,4], is thought to be the hub of appetite signals [5]. Abnormal hypothalamic functions can directly induce weight gain. For instance, hypothalamic inflammation has been linked to the progression and development of obesity [6]. In return, diet-induced obesity also disrupts the activity of hypothalamic neuronal circuits, which disturbs the regulation of body weight and food intake [7,8]. High-fat-diet (HFD)-induced obesity activates the inflammatory pathways in the hypothalamus and causes the abnormal expression of orexigenic/anorexigenic neuropeptides [9]. However, how an HFD affects the central regulation of feeding remains largely elusive.

The tripartite motif (TRIM) family is defined by the presence of a common domain structure composed of a RING finger followed by a B-box and coiled-coil domain [10] and displays E3 ubiquitin ligase activity [11]. TRIM67 is a new-found member of the TRIM protein family. Most of the research on TRIM67 has focused on studying its functions in cancers [12,13,14]. A few studies showed that TRIM67 was implicated in neuritogenesis [15,16,17]. However, the function of TRIM67 in vivo has not been well-defined and its role in the hypothalamus is completely unclear. Here, we report that the deletion of TRIM67 in the hypothalamus reduced the innervation of the sympathetic nervous system in adipose tissue and exacerbated hypothalamic inflammation under high-fat-diet exposure. We also suggest that TRIM67 could be a potential diagnosis target; a person who is deficient in TRIM67 may also be susceptible to diet-induced obesity.

## 2. Results

### 2.1. TRIM67 Is Responsive to Energy Homeostasis

**Figure 1:**
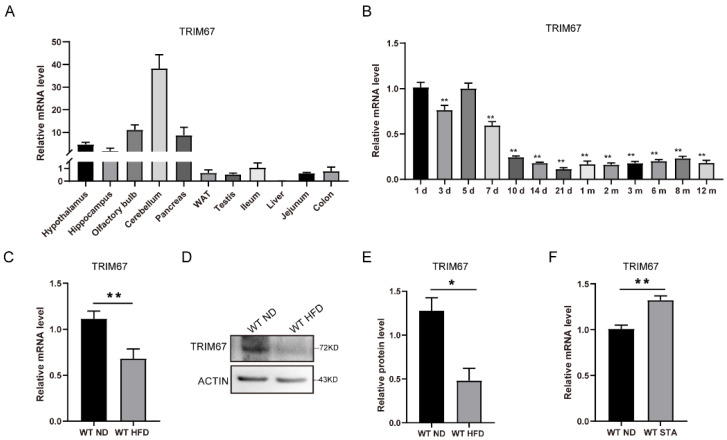
TRIM67 is responsive to energy homeostasis. (**A**) RT-qPCR analysis for the abundance of TRIM67 in different organs (*n* = 4). (**B**) The expression levels of TRIM67 in hypothalamus tissues from the different ages of WT mice (*n* = 4). (**C**) The expression of TRIM67 in hypothalamus of male mice after a 14-week high-fat diet (*n* = 4). (**D**) The protein level of TRIM67 in hypothalamus of male mice after a 14-week high-fat diet (*n* = 4). (**E**) The gray value statistical analysis of TRIM67 (*n* = 4). (**F**) The expression of TRIM67 in hypothalamus after 24 h starvation (*n* = 4). Four samples from each group were used for RT-qPCR analysis and two duplicate samples were measured. The data are presented as means ± SEM. * *p*-value < 0.05; ** *p*-value < 0.01.

The evolutionary conservation gene *TRIM67* has been reported to be rich in the nerve system [15]. Our RT-qPCR data revealed that *TRIM67* was highly expressed in brain tissue, including in the hypothalamus (Figure 1A). The expression of *TRIM67* in the hypothalamus was high within the first 7 days after birth, followed by a decline from day 10 (Figure 1B). Most interestingly, the expression of *TRIM67* in the hypothalamus was responsive to energy homeostasis. We found that the mRNA and protein levels of TRIM67 in the hypothalamus were decreased in high-fat-diet (HFD) mice compared with normal-diet (ND) mice (Figure 1C–E). The expression level of *TRIM67* increased after 24 h of starvation (Figure 1F). Given the role of the hypothalamus in defining the neural circuitry that integrates external and internal stimuli, these data suggested that TRIM67 might play a role in metabolic feedback in the hypothalamus.

### 2.2. TRIM67 Deletion Slightly Affects the Development of the Hypothalamus

**Figure 2:**
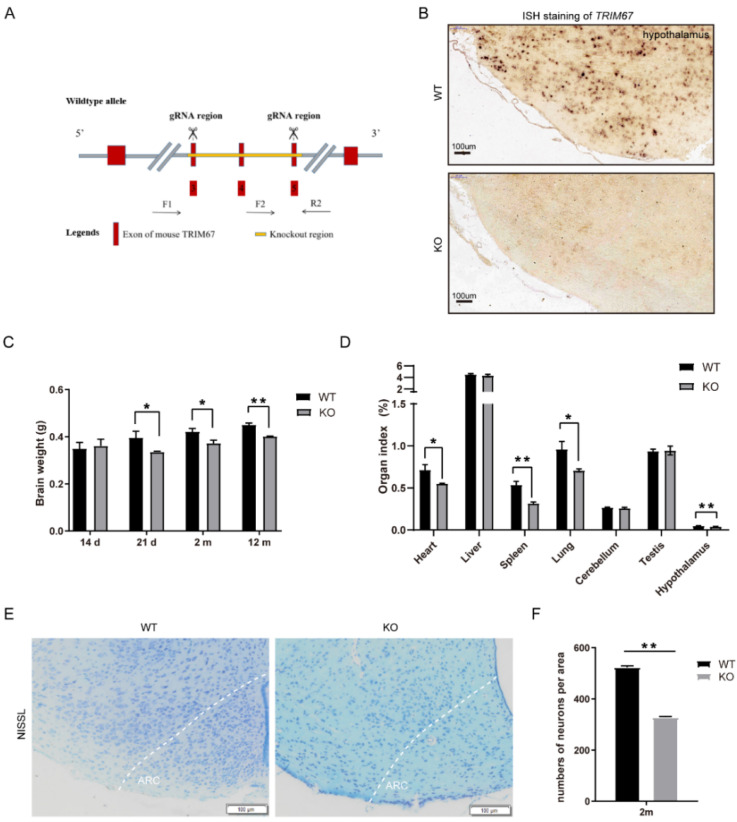
TRIM67 deletion slightly affects the development of hypothalamus. (**A**) Schematic diagram showing TRIM67 knockout using the CRISPR–Cas9 method. (**B**) ISH staining showed that *TRIM67* was completely deleted in hypothalamus. (**C**) The brain weight of WT and KO mice at 14 d, 21 d, 2 m, and 12 m (*n* = 4). (**D**) Organ indexes of 2 m WT and KO mice, including the heart, liver, spleen, lung, cerebellum, testicles, and hypothalamus (*n* = 4). (**E**) NISSL staining of 2 m WT and KO mice imaging the ARC region of hypothalamus by Olympus software (*n* = 3). (**F**) Statistical analysis was performed on the number of cells in the NISSL section of hypothalamic ARC of 2 m WT and KO mice. Four samples from each group were used for RT-qPCR analysis and two duplicate samples were measured. The data are presented as means ± SEM. * *p*-value < 0.05; ** *p*-value < 0.01.

To further elucidate the function of TRIM67 in the hypothalamus, we generated a *TRIM67* knockout mouse (TRIM67 KO) by knocking out exons 3 to 5 of the *TRIM67* gene (Figure 2A). In situ hybridization (ISH) staining showed that *TRIM67* was completely deleted in the hypothalamus (Figure 2B). The TRIM67 KO mice could be born with an expected Mendelian ratio and displayed normal postnatal viability. We found the weight of the brain was decreased in the TRIM67 KO mice (Figure 2C) and the organ index (organ index = organ weight/body weight ×100%) of the hypothalamus was also decreased compared with the WT mice (Figure 2D). To further reveal the effect of TRIM67 in the development of the hypothalamus at a morphologic level, NISSL staining was performed. We focused on the arcuate nucleus (ARC) of the mediobasal hypothalamus (MBH) because this region plays a key regulatory function in energy homeostasis [18]. We found the cell number of hypothalamic neurons was decreased in the TRIM67 KO mice (Figure 2E,F). The data above indicated that TRIM67 might regulate the normal development of the hypothalamus.

### 2.3. TRIM67 Deletion Activates AgRP-Expressing Neurons in HFD-Induced Obese Mice

**Figure 3:**
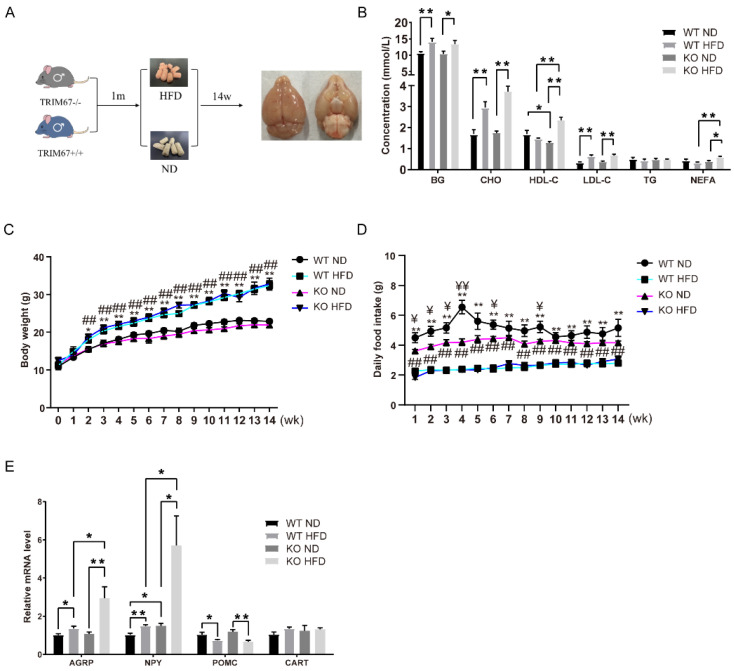
TRIM67 deletion activates AgRP-expressing neurons in HFD mice. (**A**) Schematic diagram of building an HFD mouse model. (**B**) Levels of certain serum biochemicals between groups (*n* = 4). (**C**) Sequential changes in body weight (*n* = 8). The data are presented as means ± SEM. * WT ND vs. WT HFD; # KO ND vs. KO HFD; *: *p* < 0.05; ** or ##: *p* < 0.01. (**D**) Daily food intake of mice (*n* = 8). The data are presented as means ± SEM. ¥ WT ND vs. KO ND; * WT ND vs. WT HFD; # KO ND vs. KO HFD; # or ¥: *p* < 0.05; ** or ## or ¥¥: *p* < 0.01. (**E**) The expression levels of orexigenic neuropeptides (AGRP and NPY) and anorexigenic neuropeptides (POMC) in the WT ND, WT HFD, KO ND, and KO HFD groups (*n* = 4). Four samples from each group were used for RT-qPCR analysis and two duplicate samples were measured. The data are presented as means ± SEM. * *p*-value < 0.05; ** *p*-value < 0.01.

The hypothalamus plays a critical role in diet-induced obesity (DIO) [19]. To elucidate the role of TRIM67 in DIO, 1-month-old TRIM67 KO mice and WT mice were fed a high-fat diet for 14 weeks (Figure 3A). The blood glucose (BG), blood cholesterol (CHO), and low-density lipoprotein (LDL) were higher after a high-fat-diet feed, but there was no statistical difference between the TRIM67 KO and control mice (Figure 3B). No significant change in body weight was found between the WT and TRIM67 KO mice (Figure 3C). A slightly lower food intake was detected in the TRIM67 KO mice with a normal diet, but the same food intake in the WT and TRIM67 KO mice under HFD exposure suggested that deleting TRIM67 induced a greater food intake under HFD exposure (Figure 3D). Two functionally antagonistic neuronal populations in the arcuate nucleus of the hypothalamus (ARC) sense the energy state and allow for accurate feeding behavior regulation [20]. One subset of neurons expresses the orexigenic neuropeptides agouti-related peptide (AgRP) and neuropeptide Y (NPY); the other subset expresses the anorexigenic peptides proopiomelanocortin (POMC) and cocaine and amphetamine-regulated transcript (CART). Under HFD exposure, we found the mRNA levels of AgRP and NPY were increased in the TRIM67 KO mice compared with the WT mice (Figure 3D). Although the anorexigenic genes were decreased after a high-fat diet, no statistical difference was observed after a TRIM67 deficiency (Figure 3E). Thus, the data indicated that deleting TRIM67 specifically activated AgRP-expressing neurons in HFD-induced obese mice.

### 2.4. TRIM67 Deletion Reduces Fat Sympathetic Nervous System Innervation via BDNF

**Figure 4:**
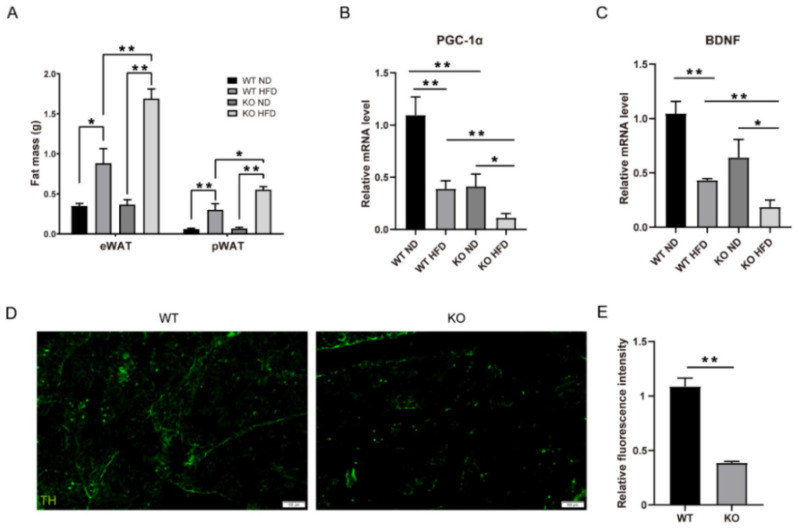
TRIM67 deletion reduces the fat sympathetic nervous system innervation via BDNF. (**A**) The weight of fat from each group (*n* = 8). (**B**) The expression levels of *PGC-1α* in WAT from each group (*n* = 4). (**C**) The expression levels of brain-derived neurotrophic factor (BDNF) in hypothalamus (*n* = 4). (**D**) The immunostaining of TH in BAT and imaging by Olympus software (*n* = 3). (**E**) Quantification of fluorescence density of TH (*n* = 3). Four samples from each group were used for RT-qPCR analysis and two duplicate samples were measured. The data are presented as means ± SEM. * *p*-value < 0.05; ** *p*-value < 0.01.

An imbalance between the energy in and the energy out can directly induce the excessive accumulation of fat [21]. We found that the fat masses of both the epididymal white adipose tissue (eWAT) and perirenal white adipose tissue (pWAT) in the TRIM67 KO mice were heavier than those in the WT mice under HFD exposure (Figure 4A). Consistent with the increased fat mass in the TRIM67 KO mice, a key transcription regulator gene, peroxisome proliferator-activated receptor γ coactivator 1α (*PGC-1α*)—which induces the expression of mitochondrial fatty acid oxidation and thermogenic genes in adipose tissue [22]—was significantly decreased in the adipose tissue of the TRIM67 KO mice (Figure 4B). This was not due to a TRIM67 deficiency in the adipose tissue of the TRIM67 KO mice because *TRIM67* expresses little in adipose tissue (Figure 1A). Adipose tissue is innervated by the sympathetic nervous system (SNS) that regulates thermogenesis in brown adipose tissue (BAT) and lipolysis in WAT [23]. Recently, a sophisticated study showed that brain-derived neurotrophic factor (BDNF), which is expressed in the paraventricular nucleus of the hypothalamus, regulated the sympathetic innervation of subcutaneous white and brown adipose tissue [24]. We hypothesized that a reduced BDNF might account for the accumulation of fat in the TRIM67 KO mice. An RT-qPCR assay showed that the mRNA of BDNF was reduced in the hypothalamus of the TRIM67 KO mice (Figure 4C). Furthermore, immunostaining with tyrosine hydroxylase (TH), which was used to mark SNS neurons, showed a significant decrease in the SNS innervation of BAT in the TRIM67 KO mice (Figure 4D,E). Therefore, the data above suggested that a TRIM67 deficiency in the hypothalamus induced fat accumulation via the BDNF–SNS axis.

### 2.5. TRIM67 Deletion Increases Hypothalamic Inflammation in HFD Mice

**Figure 5:**
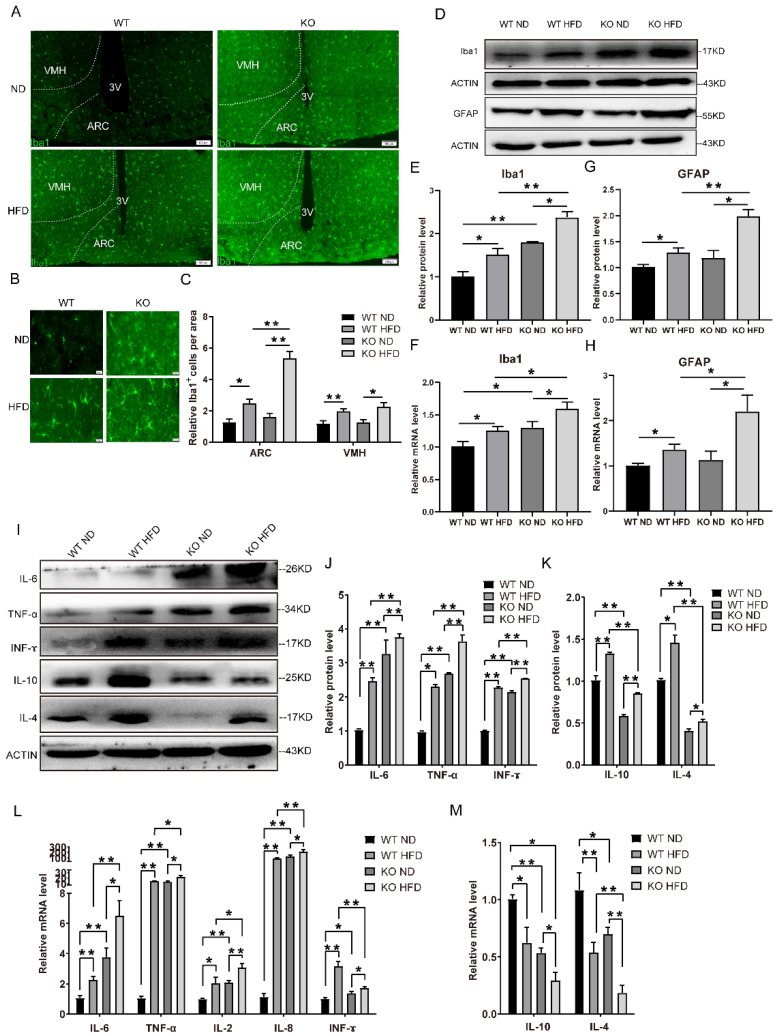
TRIM67 deletion increases hypothalamic inflammation in HFD mice. (**A**) The immunostaining of Iba1 in hypothalamus from four groups and imaging by Olympus software (*n* = 3). (**B**) The magnified images of the ARC region (*n* = 3). (**C**) The quantity of Iba1-positive cells in the ARC region (*n* = 3). (**D**) The protein levels in hypothalamus of Iba1 and GFAP were assayed by Western blots (*n* = 3). (**E**) The gray value statistical analysis of Iba1 (*n* = 3). (**F**) The mRNA level of Iba1 was assayed by RT-qPCR (*n* = 4). (**G**) The gray value statistical analysis of GFAP (*n* = 3). (**H**) The mRNA level of GFAP was assayed by RT-qPCR (*n* = 4). (**I**) The protein levels in hypothalamus of pro-inflammatory factors and anti-inflammatory factors were assayed by Western blots (*n* = 3). (**J**) The gray value statistical analysis of IL-6, TNF-α, and IFN-γ, respectively (*n* = 3). (**K**) The gray value statistical analysis of IL-10 and IL-4 (*n* = 3). (**L**) The mRNA levels of pro-inflammatory factors in hypothalamus (*n* = 4). (**M**) The mRNA levels of anti-inflammatory factors in hypothalamus (*n* = 4). Four samples from each group were used for RT-qPCR analysis and two duplicate samples were measured. The data are presented as means ± SEM. * *p*-value < 0.05; ** *p*-value < 0.01.

An HFD may trigger hypothalamic inflammation, which possibly disrupts the activity of AgRP- and POMC-expressing neurons [7,25,26]. Meanwhile, a chronic inflammatory state is known to affect several BDNF-related signaling pathways [27]. As we had observed the activation of AgRP-expressing neurons and reduced BDNF in the TRIM67 KO mice, we speculated whether hypothalamic inflammation participated in the disordered neuronal activity in the hypothalamus of the TRIM67 KO mice. Microglia and astrocytes serve the innate immune responses in the brain [28]. First, we assessed the microglia and astrocytes in the hypothalamus of the TRIM67 KO mice and controls. Immunostaining with Iba1, which is the marker of microglia, revealed a robust increase in microglia in the ARC of the TRIM67 KO mice (Figure 5A–C). The shape of the microglia in the TRIM67 KO mice was amoeba-like (Figure 5B), indicating the activation of microglia. RT-qPCR and Western blots also showed an increase in Iba1 levels in the TRIM67 KO mice compared with the WT (Figure 5D–F). Consistent with the microglia, the RT-qPCR and Western blots of the GFAP showed that the activated astrocytes were increased in the TRIM67 KO mice (Figure 5D,G,H). Microglia and astrocytes release several cytokines to respond to the inflammatory response [28]. We found that pro-inflammatory cytokines such as IL-6, TNF-α, and IFN-γ were significantly increased in the protein levels of the TRIM67 KO mice compared with the WT mice (Figure 5I,J). Similarly, the mRNA levels of pro-inflammatory cytokines such as IL-6, TNF-α, IL-2, IL-8, and IFN-γ were increased in the TRIM67 KO mice (Figure 5L). In contrast, anti-inflammatory cytokines such as IL-10 and IL-4 were decreased in the mRNA and protein levels of the TRIM67 KO mice (Figure 5I,K,M). The data above suggested that the deletion of TRIM67 induced a stronger hypothalamic inflammation in the HFD mice.

### 2.6. TRIM67 Deletion Activates the NF-κB Pathway

**Figure 6:**
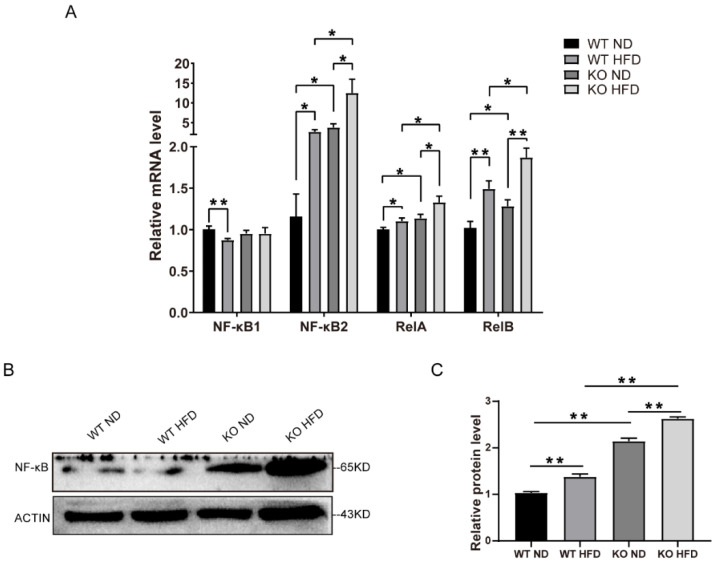
TRIM67 deletion activates NF-κB pathway. (**A**) The mRNA levels of NF-κB in hypothalamus (*n* = 4). (**B**) The protein levels of NF-κB in hypothalamus (*n* = 3). (**C**) The gray value statistical analysis of NF-κB (*n* = 3). Four samples from each group were used for RT-qPCR analysis and two duplicate samples were measured. The data are presented as means ± SEM. * *p*-value < 0.05; ** *p*-value < 0.01.

The transcription factor NF-κB is a key regulator of immunity and can induce the expression of pro-inflammatory cytokines in both microglia cells and astrocytes [28,29]. We further investigated a possible connection between the NF-κB pathway and the dysregulation of the energy balance in the hypothalamus. The NF-κB family of transcription factors, NF-κB2 (p52), RelA, and RelB, were increased in the mRNA levels of the TRIM67 KO mice compared with the controls (Figure 6A). The Western blots also revealed an increase in the NF-κB of the TRIM67 KO mice (Figure 6B,C). Thus, the data demonstrated that TRIM67 deletion increased hypothalamic inflammation by activating the NF-κB pathway under HFD exposure.

### 2.7. TRIM67 Deletion Induces Apoptosis in the Hypothalamus

**Figure 7:**
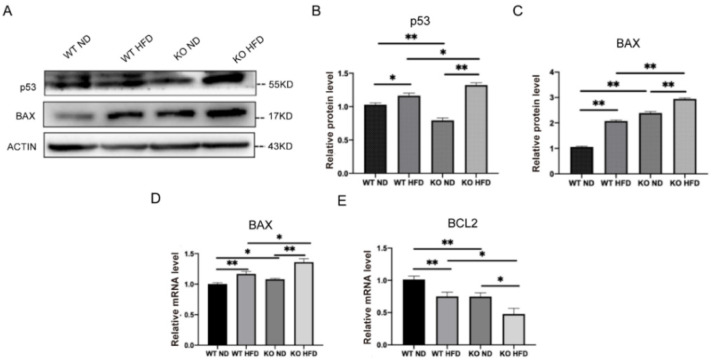
TRIM67 deletion induces apoptosis in hypothalamus. (**A**) The protein levels of BAX and P53 in hypothalamus (*n* = 3). (**B**,**C**) The gray value statistical analysis of BAX and P53, respectively (*n* = 3). (**D**) The expression level of BAX in hypothalamus (*n* = 4). (**E**) The expression level of BCL2 in hypothalamus (*n* = 4). Four samples from each group were used for RT-qPCR analysis and two duplicate samples were measured. The data are presented as means ± SEM. * *p*-value < 0.05; ** *p*-value < 0.01.

As the inflammatory signal can lead to the activation of apoptotic signaling pathways [30], we evaluated the effect of TRIM67 deletion on the induction of apoptosis in the hypothalamus under HFD exposure. The tumor suppressor protein P53 triggers cell apoptosis. We found that P53 was increased in the TRIM67 KO mice under HFD exposure assayed by Western blots (Figure 7A,B). P53 can regulate the transcription of pro-apoptotic protein BAX; we found that BAX was increased in the protein and mRNA levels of the TRIM67 KO mice (Figure 7A,C,D). In contrast, the expression of anti-apoptotic protein BCL2 was decreased in the TRIM67 KO mice compared with the control mice (Figure 7E). Combined with the decreased number of hypothalamic neurons we observed before (Figure 2D,E), the data suggested that a TRIM67 deficiency could induce a higher level of apoptosis in the hypothalamus under HFD exposure.

## 3. Discussion

As the central regulator of energy homeostasis, the hypothalamus receives afferent and efferent signals from the brainstem and peripheral tissue and forms a complex appetite regulation circuit to effectively regulate food intake and energy expenditure. Growing evidence shows that insulin, leptin, and ghrelin, for example, act on the neurons in the ARC to regulate feeding behavior [31,32], but our understanding of how environmental signaling affects hypothalamic functions remains inadequate. In this study, we identified that the hypothalamic TRIM67 level was responsive to energy homeostasis. The expression level of TRIM67 was reduced under HFD exposure whereas it was increased after starvation. With the TRIM67 KO mice, we found that a TRIM67 deficiency increased their susceptibility to DIO and the fat mass was higher in TRIM67 KO mice. The data suggested that TRIM67 played a role in the hypothalamus functions of regulating energy homeostasis. Although we did not know the molecular mechanism behind TRIM67 and energy homeostasis, our last work—in which the TRIM67 expression was linked to PGC-1α [33]—provided a potential relevance.

The imbalance of energy homeostasis initiated by the consumption of a high-fat diet represents a key step in the development of obesity, in which the hypothalamus plays a critical role. Using the TRIM67 KO mice, we found that a TRIM67 deficiency exacerbated fat accumulation and increased the activity of AgRP-expressing neurons under HFD exposure. AgRP-expressing neurons are critical for producing a central representation of hunger [34]. Activated AgRP-expressing neurons not only stimulate a voracious food intake [35] by releasing AgRP, NPY, and GABA [36], but also activated AgRP-expressing neurons increase the respiratory exchange ratio (RER), indicating elevated carbohydrate utilization, reduced lipolysis [37,38,39,40], and the suppression of the thermogenic program of white fat [41]. Furthermore, we found BDNF was reduced in the hypothalamus of the TRIM67 KO mice. On the one hand, BDNF directly administrates the ventromedial nucleus or paraventricular nucleus (PVN) in the hypothalamus and positively affects energy expenditure [42,43]. On the other hand, BDNF regulates the sympathetic innervation of subcutaneous white and brown adipose tissue [24]. Consistent with a reduced BDNF, we found that the fat mass was increased and SNS innervation was decreased in the adipose tissue of the TRIM67 KO mice. Although we detected increased fat accumulation in the TRIM67 KO mice fed with the HFD, no significant change in body weight was found between the WT and TRIM67 KO mice. In our opinion, widespread weight loss in multiple organs was in this mouse model, which may be a consequence of the whole-body knockout we used, offsetting the body weight gain from the fat gain.

In the last few decades, evidence has highlighted the role of hypothalamic inflammation in DIO [44,45]. The overconsumption of a fat-rich diet results in acute changes to hypothalamic inflammatory responses [6]. In turn, hypothalamic inflammation results in an uncoupling between food intake and energy expenditure, leading to overeating and further weight gain [6,45]. An HFD-induced activation of non-neuronal cells such as microglia and astrocytes produces inflammatory reactions in the hypothalamus [26,44,46]. We found that the level of hypothalamic inflammation was higher in the TRIM67 KO mice under HFD exposure, including higher levels of pro-inflammatory cytokines and lower levels of anti-inflammatory cytokines. Furthermore, cytokines can lead to a reduction in growth factors such as BDNF [47]. HFD consumption induces hypothalamic inflammation via the activation of nuclear factor-κB (NF-κB) pathways [2,48]. TRIM67 was reported to negatively regulate the NF-κB signaling pathway by competitively binding beta-TrCP to IkBa [49]. In this study, we found that an HFD induced the expression of NF-κB in the hypothalamus and a TRIM67 deficiency exacerbated the increase in the NF-κB expression. In addition, prolonged inflammation leads to the apoptosis of hypothalamic neurons and anorexigenic POMC-expressing neurons are the main targets of inflammation-induced apoptosis [30,50,51]. We also found that a TRIM67 deficiency induced a higher level of apoptosis in the hypothalamus under HFD exposure.

## 4. Materials and Methods

### 4.1. Animals

In the current work, TRIM67 heterozygous (HET, TRIM67+/−) C57BL/6 male and female mice were procured from Cyagen Biosciences Inc. (Guang Zhou, China). One male mouse and two female mice were kept in a standard cage (485 mm × 350 mm × 200 mm) and placed in an environmentally controlled room (18 to 22 °C, 40 to 70% humidity, 12 h light). After two generations of reproduction, all F3 offspring (wild-type (WT, TRIM67+/+), homozygous (KO, TRIM67−/−), and HET) DNA were extracted following the standard method from the tail and PCR amplified using 2 × Rapid Taq Master Mix (Vazyme, Nanjing, China), followed by an imaging analysis using a GelDoc system (Bio-Rad, Hercules, CA, USA) equipped to identify the genotype. For further information, see Table 1. We then randomly divided sixteen four-week-old WT male mice and sixteen four-week-old KO male mice into two groups, which were fed with either a normal diet (WT ND and KO ND) or a high-fat diet (WT HFD and KO HFD) for fourteen weeks, respectively. The experimental procedures were approved by the Animal Care and Use Committee of the College of Veterinary Medicine, Sichuan Agricultural University, China.

### 4.2. Measurement of Blood Fat

All mice fasted overnight before slaughter and were anesthetized via an intraperitoneal injection of 10% chloral hydrate. We obtained the tail blood, then the blood glucose (BG) levels were immediately measured using an automatic blood glucose meter (YUWELL, Jiangsu, China). The heart blood was collected using a disposable syringe and then centrifuged at 3000 rpm at 4 °C for 5 min to obtain the serum. Finally, the serum levels of the blood fat, including triglyceride (TG), cholesterol (TC), high-density lipoprotein cholesterol (HDL-C), and low-density lipoprotein cholesterol (LDL-C), were measured using a commercial kit based on enzymatic colorimetry (Jiancheng, Nanjing, China).

### 4.3. RT-qPCR

The total RNA from each sample was obtained using RNAiso Plus Reagent (Invitrogen, Carlsbad, CA, USA) according to the standard method. An Agilent Bioanalyzer (Agilent Technologies, Santa Clara, CA, USA) was used for the quality test and only eligible RNA (A160/A180 = 1.6~1.8, concentration > 200 ng/μL) was used for the later trial. A reverse transcription of the mRNA was performed using RT Easy^TM^ II (with gDNase) (FOREGENE, Chengdu, China). In addition, the RT-qPCR was performed in triplicate using Real-Time PCR Easy^TM^ SYBR Green I (FOREGENE, Chengdu, China) on a CFX96 instrument (Bio-Rad, Hercules, CA, USA). The relative levels of mRNA were calculated using the 2^−ΔΔCt^ method. *β-Actin* was used as the internal reference for the mRNA quantification. The sequence information is listed in Table 2.

### 4.4. Western Blots

The total protein was extracted using a commercial kit (Sigma, Germany) following the manufacturer’s protocol. The concentration of the protein was then tested using a Bradford protein assay kit (Beyotime, Shanghai, China); only protein meeting the criteria (concentration > 1 mg/mL) were used for the further trial. Briefly, the protein was resolved via SDS-PAGE and then transferred to a PVDF membrane, followed by a sealing fluid. The membranes were incubated with the corresponding primary antibodies at 4 °C overnight and subsequently incubated with the secondary antibodies at room temperature for 1 h. The membranes were subjected to a chemiluminescence reagent to detect immunoreactivity. A GelDoc system (Bio-Rad, Hercules, CA, USA) was used to capture the images. Protein β-actin was used as an internal control.

### 4.5. Histological Staining

The brains were fixed in a 4% paraformaldehyde solution and embedded in paraffin. The 5 μm sections were mounted on slides and stained with NISSL according to the manufacturer’s instructions (G1432, Solarbio, Beijing, China). The images were captured using an inverted microscope (Olympus, Tokyo, Japan).

### 4.6. Immunofluorescence Staining

The immunofluorescence of the SNS neurons was performed following the method described in a previous work [24]. Briefly, the hypothalamus sections were mounted in an optimal cutting temperature compound (OCT) and 20 µm-thick coronal sections were cut using a cryostat (CM1850, Leica, Nussloch, Germany). In addition, the adipose tissue was defatted with 20%, 40%, 60%, 80%, and 100% methanol/B1n buffer for 30 min before being frozen and then treated with DCM three times for 30 min, 1 h, and 30 min, respectively. It was then successively treated with 100%, 100%, 80%, 60%, 40%, and 20% methanol/B1n buffer for 30 min. After a 30 min treatment with the B1n buffer, it was treated with the new B1n buffer for the whole night. The above degreasing steps were all on ice on an orbital shaker set at ~100 rpm. The tissue was then sectioned at 40 µm using a Leica microtome. The primary antibody TH (1:400; AB152, Millipore) was used for the staining.

### 4.7. Statistical Analysis

All data were presented as means ± SEM. GraphPad software (GraphPad Software Inc., La Jolla, CA, USA) was used for the statistical analysis and the differences between the groups were determined by a Student’s *t*-test. The differences were considered to be statistically significant at a *p*-value < 0.05.

## 5. Conclusions

In summary, in this study we revealed that a hypothalamic TRIM67 deficiency exacerbated hypothalamic inflammation and damage, which increased the susceptibility of mice to DIO. We also showed that TRIM67 may be implicated in the regulation of fat accumulation through a BDNF-affected fat sympathetic nervous system innervation. These results provide further evidence for a TRIM67 function in energy metabolism and TRIM67 as a diagnostic and therapeutic target for obesity.

## Figures and Tables

**Table 1:** The primers used for genotype identification.

	Primer	Sequence (5′-3′)
KO	F	GATGATAGCCATGTAATGCCCACC
	R	CCGTGATATGCTT-GCCACAGGTTC
WT	F	GATGA-TAGCCATGTAATGCCCACC
	R	TGCCGTTTTCCCCTTCTAAATCAG

**Table 2:** The primers used for the RT-qPCR analysis.

Gene	Sequence (5′-3′)
*β-Actin*	F: AGAGGGAAATCGTGCGTGAC
	R: CAATAGTGATGACCTGGCCGT
*IL-6*	F: CTTCCATCCAGTTGCCTTCTTG
	R: AATTAAGCCTCCGACTTGTGAAG
*IL-8*	F: GTGGCTTTGCCGTGCAATAA
	R: TAGAGGGCATGCCAGAGCTA
*TNF-α*	F: ACGGCATGGATCTCAAAGAC
	R: GTGGGTGAGGAGCACGTAG
*TRIM67*	F: GGCGAAGGAGTTTCTGGTTC
	R: TAGCTTCAGGGTGCAGTGATT
*IL-4*	F: CTTCCAAGGTGCTTCGCATA
	R: GATGAATCCAGGCATCGAAA
*IL-10*	F: AAGGGTTACTTGGGTTGCCA
	R: CCTGGGGCATCACTTCTACC
*TGF-β*	F: GTGTGGAGCAACATGTGGAACTCTA
	R: CGCTGAATCGAAAGCCCTGTA
*IL-17*	F: CTGATCAGGACGCGCAAAC
	R: TCGCTGCTGCCTTCACTGTA
*IL-2*	F: CCTGAGCAGGATGGAGAATTACA
	R: TCCAGAACATGCCGCAGAG
*POMC*	F: ATAGATGTGTGGAGCTGGTG
	R: GGCTGTTCATCTCCGTTG
*CART*	F: GCGCTATGTTGCAGATCGAA
	R: TCACACAGCTTCCCGATCCT
*AGRP*	F: CAGACCGAGCAGAAGAAG
	R: GACTCGTGCAGCCTTACA
*NPY*	F: CCGCTCTGCGACACTACAT
	R: TGTCTCAGGGCTGGATCTCT
*LEP*	F: TGAGCAGGCGTGCCATC
	R: GTACCCGTCAGTTTCACATGATATA
*IGFBP2*	F: GCGGGTACCTGTGAAAAGAG
	R: CCTCAGAGTGGTCGTCATCA
*GFAP*	F: CAACGTTAAGCTAGCCCTGGACAT
	R: CTCACCATCCCGCATCTCCACAGT
*IBA1*	F: CTTTTGGACTGCTGAAGGC
	R: GTTTCTCCAGCATTCGCTTC
*PCK1*	F: ATGTGTGGGCGATGACATTGC
	R: AACCCGTTTTCTGGGTTGATAG
*IFN-* *ɤ*	F: CTGGAGGAACTGGCAAAAGGATGG
	R: GACGCTTATGTTGTTGCTGATGGC
*NF-κB1*	F: GAAATTCCTGATCCAGACAAAAAC
	R: ATCACTTCAATGGCCTCTGTGTAG
*NF-κB2*	F: CTGGTGGACACATACAGGAAGAC
	R: ATAGGCACTGTCTTCTTTCACCTC
*RelA*	F: CTTCCTCAGCCATGGTACCTCT
	R: CAAGTCTTCATCAGCATCAAACTG
*RelB*	F: CTTTGCCTATGATCCTTCTGC
	R: GAGTCCAGTGATAGGGGCTCT
*TRIM67*	F: GGCGAAGGAGTTTCTGGTTC
	R: TAGCTTCAGGGTGCAGTGATT
*BDNF*	F: GACAAGGCAACTTGGCCTAC
	R: ACTGTCACACGCTCAGC
*HSL*	F: CCAGCCTGAGGGCTTACTG
	R:CTCCATTGACTGTGACATCTCG
*Adcyap1*	F: ACCATGTGTAGCGGAGCAAG
	R: CTGGTCGTAAGCCTCGTCT
*Ppargc1a*	F: TATGGAGTGACATAGAGTGTGCT
	R: CCACTTCAATCCACCCAGAAAG

## Data Availability

Source data are provided in this paper and are available from the corresponding author upon reasonable request.
